# Human Bone Marrow Mesenchymal Stem Cells Promote Gastric Cancer Growth via Regulating *c-Myc*

**DOI:** 10.1155/2018/9501747

**Published:** 2018-07-18

**Authors:** Bin Chen, Jing Yu, Qianqian Wang, Yuanyuan Zhao, Li Sun, Changgen Xu, Xiangdong Zhao, Bo Shen, Mei Wang, Wenrong Xu, Wei Zhu

**Affiliations:** ^1^School of Medicine, Jiangsu University, Zhenjiang, Jiangsu, China; ^2^The Xuyi People's Hospital, Xuyi, Jiangsu, China; ^3^Zhenjiang Provincial Blood Center, Zhenjiang, Jiangsu, China; ^4^Department of Oncology, Jiangsu Cancer Hospital Affiliated to Nanjing Medical University, Nanjing, Jiangsu, China

## Abstract

The clinical application of human bone marrow mesenchymal stem cells (hBM-MSCs) has generated a great deal of interest because of their potential use in regenerative medicine and tissue engineering. However, safety concerns over hBM-MSCs limit their clinical application. In this study, we observed that hBM-MSC-conditioned medium (hBM-MSC-CM) promotes gastric cancer development via upregulation of c-Myc. Our results showed that c-Myc was upregulated in MGC-803 and BGC-823 cells after hBM-MSC-CM treatment. Moreover, we found that the c-Myc inhibitor JQ1 and c-Myc siRNA decreased the expression of c-Myc in hBM-MSC-CM-treated tumor cells *in vitro*. Additionally, hBM-MSC-CM enhanced the migration and glucose uptake of gastric cancer cells. *In vivo* studies showed that JQ1 inhibited hBM-MSC-CM-induced gastric cancer growth. These results indicated that hBM-MSC-CM induced gastric cancer growth via upregulation of c-Myc, which may be a potential risk factor and/or a therapeutic target for clinical applications.

## 1. Introduction

Due to their multidirectional differentiation capacity, mesenchymal stem cells (MSCs) have been widely used in tissue engineering and cell replacement therapy [1]. However, studies have increasingly demonstrated that human bone marrow MSCs (hBM-MSCs) can promote tumor growth and metastasis [2, 3], making safety of hBM-MSCs in clinical applications controversial.

Because of their promise for clinical applications, a variety of models have been used to prove the safety and effectiveness of hBM-MSCs [4, 5]. Some previous clinical studies have concluded that hBM-MSCs do not pose an obvious risk of tumorigenesis when used to treat cartilage injuries or other diseases [6, 7]. However, other studies have found that hBM-MSCs promote tumor proliferation, migration, and stemness *in vitro* and that hBM-MSCs promote tumor development *in vivo* [3, 8, 9]. Therefore, there is an urgent need to identify the factors from hBM-MSCs that promote tumor growth.

In this study, we found that hBM-MSC-CM caused gastric cancer cells to upregulate c-Myc expression, which is a well-known oncogene that is involved in tumor initiation and development. Abnormal c-Myc activation is responsible for a range of human cancers, including neuroblastoma [10], lung carcinoma [11], and gastric carcinoma [12]. By promoting c-Myc expression, hBM-MSC-CM increased the metabolism, migration, and proliferation of gastric cancer cells. Furthermore, we showed that the c-Myc inhibitor JQ1 inhibits the tumor-promoting effects of hBM-MSC-CM. Thus, we show that hBM-MSC-CM can upregulate c-Myc expression in gastric cancer cells, which may be a key factor in carcinogenesis and, therefore, a potential target for cancer prevention.

## 2. Materials and Methods

### 2.1. Xenograft Tumor Model

This study and its consent procedure were approved by the local ethics committee of Jiangsu University (Jiangsu, China). BALB/c-nu/nu male mice (*n* = 30; aged 4-5 weeks) were purchased from SLAC Laboratory Animal (Shanghai, China) and maintained in pathogen-free conditions with sterilized chow and autoclaved water. The animals were randomly divided into five groups (*n* = 6 mice per group). MGC-803 cells were treated with either Dulbecco's modified Eagle's medium (DMEM; Gibco/Life Technologies, Carlsbad, CA, USA), dimethyl sulfoxide (DMSO; Sigma-Aldrich, St. Louis, MO, USA), hBM-MSC-conditioned media (hBM-MSC-CM), JQ1 (0.8 *μ*M), or hBM-MSC-CM + JQ1 for 48 h. In the hBM-MSC-CM + JQ1 group, MGC-803 cells were pretreated with JQ1 for 4–6 h, washed three times with phosphate-buffered saline (PBS), and then treated with hBM-MSC-CM + JQ1. Then, subcutaneous xenografts were established by injection of 2 × 10^6^ MGC-803 cells in 200 *μ*L of PBS per mouse. Once tumors formed, tumor sizes were measured using Vernier calipers and tumor volumes were calculated using the following formula: tumor volume = length × width^2^/2. The experiment was stopped on day 30, when all mice were euthanized.

### 2.2. Cell Culture

Healthy donor-derived bone marrow cells were collected at the Affiliated Hospital of Jiangsu University, and all protocols were approved by the local ethics committee of the Affiliated Hospital of Jiangsu University, China. Additionally, informed consent was received from all donors. The bone marrow cells were diluted in an equal volume of PBS, isolated with 1.077 g/mL Ficoll, and centrifuged at 800 ×g for 20 min. The cells were then rinsed with PBS and cultured in DMEM containing 10% fetal bovine serum (FBS; Gibco) at 37°C in a humidified incubator infused with 5% CO_2_; adherent cells were collected after 5 days. The human gastric cancer cell lines MGC-803 and BGC-823 and normal line GES-1 were purchased from the Cell Bank of Type Culture Collection of Chinese Academy of Sciences (Beijing, China) and were maintained in DMEM supplemented with 10% FBS at 37°C in 5% CO_2_.

### 2.3. Preparing hBM-MSC-CM and Coculture with Gastric Cancer Cells

When hBM-MSCs had grown to 70% confluency, they were washed with PBS and incubated with fresh medium for 48 h. Then, the supernatant was collected, filtered through a 0.22 *μ*m filter, and diluted at 1 : 1 with DMEM supplemented with 10% FBS (hBM-MSC-CM). MGC-803 and BGC-823 cells were washed with PBS and then treated with DMEM, DMSO, hBM-MSC-CM, JQ1 (0.8 *μ*M), or hBM-MSC-CM + JQ1 at 37°C in 5% CO_2_ for 48 h prior to being collected for use in subsequent experiments.

### 2.4. Transwell Migration Assay

Transwell migration assays were performed to evaluate the effect of JQ1 or c-Myc knockdown on the hBM-MSC-CM-induced migration of gastric cancer cells. In the experiment using JQ1, 1 × 10^5^ MGC-803 or BGC-823 cells were plated in six-well plates (Corning Inc., Corning, NY, USA) and then treated with DMEM, DMSO, hBM-MSC-CM, JQ1 (0.8 *μ*M), and hBM-MSC-CM + JQ1. In the experiment using c-Myc knockdown, the cells after c-Myc knockdown were cocultured with hBM-MSC-CM. After 48 h, the cells were collected by centrifugation (800 rpm/min) and 8 × 10^4^ cells were seeded in the upper wells of a Transwell chamber in 200 *μ*L of serum-free DMEM. The lower compartment of the chamber was filled with 500 *μ*L DMEM supplemented with 10% FBS. The cells were incubated for 8 h, and after incubation, we used a cotton swab to remove the cells that did not migrate. Migrated cells were fixed with 4% formaldehyde for 30 min, stained with crystal violet, and then photographed. For quantitation, three random fields from each well were counted under a microscope (Ti-S; Nikon, Tokyo, Japan), and each experiment was independently repeated in triplicate.

### 2.5. Glucose Uptake Assay

Glucose uptake assays were performed to evaluate the effect of hBM-MSC-CM on the glucose utilization of gastric cancer cells. Briefly, 1 × 10^5^ MGC-803 or BGC-823 cells were plated in six-well plates (Corning Inc.) and then treated with DMEM, DMSO, hBM-MSC-CM, JQ1 (0.8 *μ*M), and hBM-MSC-CM + JQ1. After 48 h, the cells were collected by centrifugation (800 rpm/min) and 1 × 10^6^ cells/mL were seeded in 48-well plates and incubated for 8 h in DMEM. Then, a clinical chemistry analyzer (Xunda, XD811, Shanghai, China) and the hexokinase method were used to detect supernatant glucose concentrations.

### 2.6. Cell Viability Assay

We performed 3-[4,5-dimethylthiazol-2yl] diphenyltetrazolium bromide (MTT) assays to assess the half maximal inhibitory concentration (IC50) of JQ1. Cells were seeded into 96-well plates (Corning Inc.) at a density of 2 × 10^3^ per well and incubated overnight at 37°C in 5% CO_2_. Then, the plates were incubated with DMSO or different JQ1 concentrations (0.4 and 0.8 *μ*M) and cultured for 24, 48, or 72 h. MTT (5 mg/mL) was added to the cells for 4 h at 37°C. After 4 h, we added DMSO to terminate the reaction; results were determined by measuring optical density at 490 nm with a multiwell plate reader (FLx800, BioTek, Winooski, VT, USA). Each experiment was repeated three times.

### 2.7. Western Blot Analysis

Total proteins were extracted from cells using RIPA lysis buffer (Invitrogen, Carlsbad, CA, USA). After determining protein concentration, 20 *μ*g of total protein from each sample was separated with 12% sodium dodecyl sulfate polyacrylamide gel electrophoresis and then transferred to polyvinylidene fluoride membranes (Millipore, Billerica, MA, USA). The membranes were blocked with 5% nonfat milk and incubated with anti-human c-Myc (dilution, 1 : 300; 10057-1-AP, Proteintech, Chicago, IL, USA), Bcl-2 (dilution, 1 : 200; 2870, Cell Signaling Technology, USA), Bax (dilution, 1 : 200; BS2538, Bioworld, USA), cyclin-D1 (dilution, 1 : 500; BS1741, Bioworld, USA), and Glut1 (dilution, 1 : 1000; FO6231163, Wanleibio, Shenyang, China) antibodies overnight at 4°C and then incubated with goat anti-mouse (dilution, 1 : 2000; CW0102, CWBIO, Beijing, China) and goat anti-rabbit (dilution, 1 : 2000; CW0103, CWBIO, Beijing, China) secondary antibodies for 1 hour at 37°C. The blots were visualized using the enhanced chemiluminescent detection system (Amersham, Amersham, UK) and analyzed using Image-Pro Plus version 5.1 (Media Cybernetics Inc., Rockville, MD, USA).

### 2.8. Cell Cycle Analysis

The effects of hBM-MSC-CM and JQ1 on cell cycle distributions were measured using a flow cytometer (FACSCalibur, BD Biosciences, Franklin Lakes, NJ, USA). Briefly, 1 × 10^5^ cells per well were seeded into six-well plates, incubated overnight, and then treated with DMEM, DMSO, hBM-MSC-CM, JQ1 (0.8 *μ*M), and hBM-MSC-CM + JQ1. After 48 h, 1 × 10^6^ cells per well were collected by centrifugation and stained with propidium iodide in PBS for 30 min at 4°C in the dark before being analyzed with flow cytometry.

### 2.9. Colony Formation Assay

MGC-803 or BGC-823 cells were treated with DMEM, DMSO, hBM-MSC-CM, JQ1, and hBM-MSC-CM + JQ1 for 48 h, and then cells were seeded at 500 cells per well in 6 cm well plates in triplicate. After 7 days of growth, the cells were washed with PBS, fixed with 4% paraformaldehyde, and stained with crystal violet. We counted the number of colonies per well under a dissecting microscope.

### 2.10. *c-Myc* Knockdown

c-Myc siRNA (50 nM) (5′-GGACTATCCTGCTGCCAAG-3′) and negative control (NC) (50 nM) were purchased from Guangzhou RiboBio. siRNA were transfected into MGC-803 with Lipofectamine® 2000 reagent (Invitrogen; Thermo Fisher Scientific Inc.) according to the instructions.

### 2.11. Statistical Analysis

All data analyses were performed using GraphPad Prism 6 (Graph Software, La Jolla, CA, USA). Differences between groups were analyzed using one-way analysis of variance. The Kruskal–Wallis *H* test was used to analyze differences between *in vivo* tumor growths. A *P* value < 0.05 was considered statistically significant.

## 3. Results

### 3.1. HBM-MSC-CM Increased c-Myc Expression in Gastric Cancer Cells

**Figure 1:**
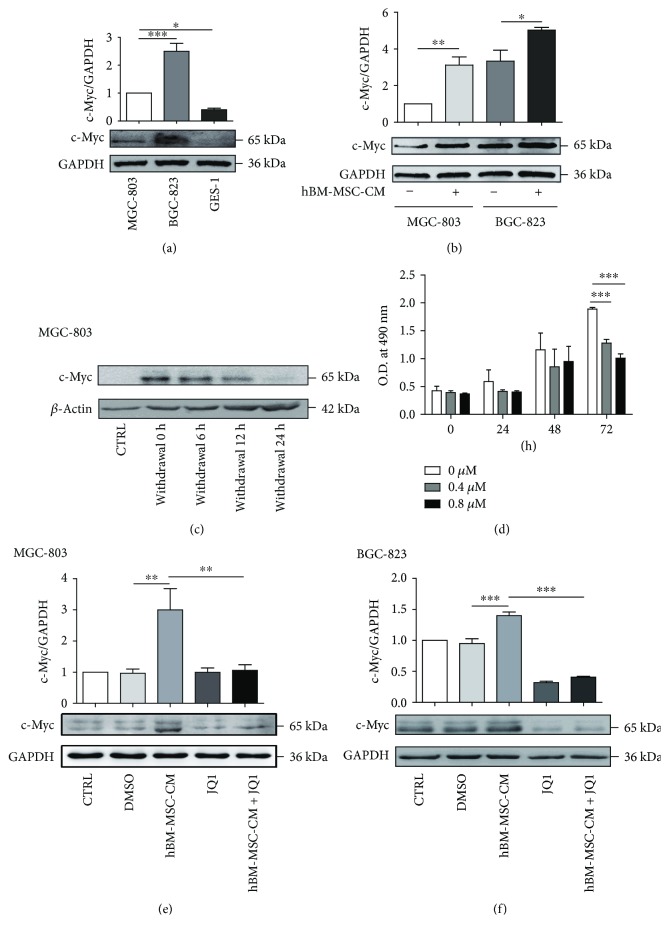
hBM-MSC-CM increased c-Myc expression in gastric cancer cells. (a) c-Myc protein levels in MGC-803, BGC-823, and GES-1 were detected by Western blot analysis. ^∗^*P* < 0.05, ^∗∗∗^*P* < 0.001. (b) c-Myc levels after 48 h hBM-MSC-CM treatment. ^∗^*P* < 0.05, ^∗∗^*P* < 0.01 (c) c-Myc protein levels maintained after withdrawal of hBM-MSC-CM treatment were detected by Western blot analysis. (d) MGC-803 cells were treated with 0.4 and 0.8 *μ*M JQ1 for 24, 48, and 72 h. ^∗∗∗^*P* < 0.001. (e, f) Compared with hBM-MSC-CM, hBM-MSC-CM + JQ1 decreased c-Myc expression in MGC-803 or BGC-823 cells. ^∗∗^*P* < 0.01, ^∗∗∗^*P* < 0.001.

The c-Myc oncogene has been reported to play important roles in gastric cancer; thus, we examined c-Myc levels in the gastric cancer cell lines MGC-803 and BGC-823 and normal line GES-1 with Western blot. GES-1 cells showed the lowest c-Myc expression, and the BGC-823 cells showed the highest c-Myc expression (Figure 1(a)). Next, we investigated whether treating the gastric cancer cells with hBM-MSC-CM for 48 h affected c-Myc protein levels. Compared with the untreated groups, c-Myc levels were increased in both MGC-803 and BGC-823 cells after hBM-MSC-CM treatment (Figure 1(b)). The upregulation of c-Myc expression in MGC-803 cells could maintain 12 hours after withdrawal of hBM-MSC-CM (Figure 1(c)). JQ1 has been shown to have antiproliferative effects in many cancers, primarily through inhibition of c-Myc [13]. MTT assays showed that 0.8 *μ*M JQ1 for 72 h has significantly antiproliferative effects on MGC-803 (Figure 1(d)). Then, we used JQ1 at 0.8 *μ*M concentration in the following experiment. Western blot analysis indicated that hBM-MSC-CM treatment can significantly increase the expression level of c-Myc protein, and the effect of hBM-MSC-CM upregulation of c-Myc in MGC-803 and BGC-823 cells can also be suppressed by JQ1 (Figures 1(e) and 1(f)).

### 3.2. JQ1 Inhibited the Gastric Cancer Cell Proliferation *In Vitro*

**Figure 2:**
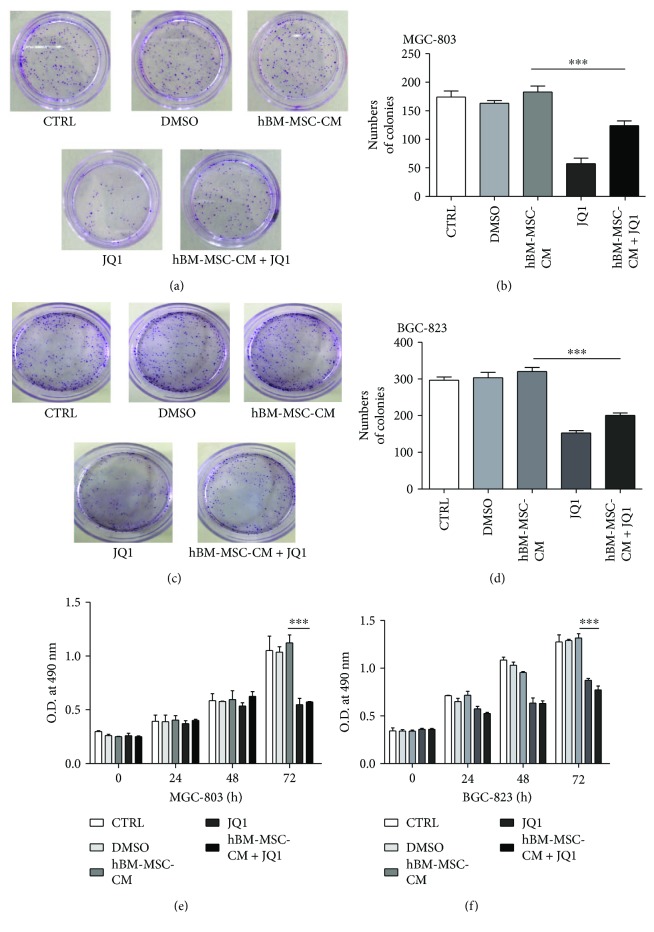
JQ1 inhibited the gastric cancer cell proliferation *in vitro*. (a, c) JQ1 reduced MGC-803 or BGC-823 colony formation rates with and without hBM-MSC-CM. (b, d) The colony formation rate of the hBM-MSC-CM + JQ1 group was lower than that of the hBM-MSC-CM group. ^∗∗∗^*P* < 0.001 by a one-way analysis of variance. (e, f) MTT assays showed that JQ1 decreased the proliferation rate of MGC-803 or BGC-823 cells compared with the hBM-MSC-CM group. ^∗∗∗^*P* < 0.001 by a one-way analysis of variance.

The effect of hBM-MSC-CM and hBM-MSC-CM + JQ1 on the proliferation of gastric cancer cells was analyzed with colony formation and MTT assays. The colony formation assays showed that the hBM-MSC-CM + JQ1 group formed fewer colonies than the hBM-MSC-CM group (*P* < 0.001, Figures 2(a)–2(d)). Consistent with the colony formation assays, MTT assays showed that JQ1 inhibited the proliferation rate of MGC-803 and BGC-823 cells (*P* < 0.001, Figures 2(e) and 2(f)).

### 3.3. Effect of hBM-MSC-CM on Cell Cycle Progression and Apoptosis

**Figure 3:**
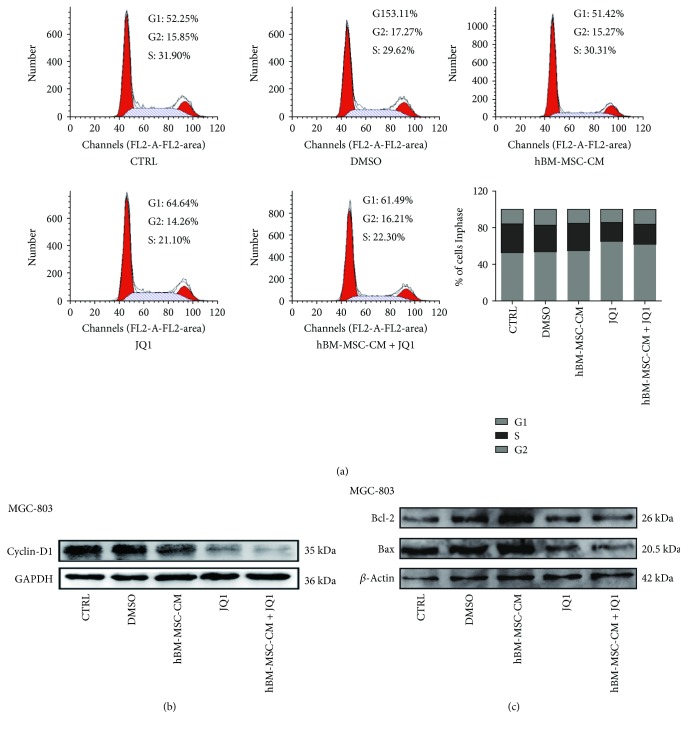
Effect of hBM-MSC-CM on cell cycle progression and apoptosis in gastric cancer cells. (a) hBM-MSC-CM + JQ1 induced a G1 arrest compared with the hBM-MSC-CM group; the changes in cell cycle distribution were not significant after three experiments. (b) Western blot analysis of cyclin-D1 expression in hBM-MSC-CM + JQ1-treated MGC-803 cells. (c) Western blot analysis of Bcl-2 and Bax expression in hBM-MSC-CM + JQ1-treated MGC-803 cells.

Cell cycle analysis revealed that JQ1 treatment increased the percentage of G1-phase cells from 51.42% to 61.49%, while the fraction of S-phase cells decreased from 30.31% to 22.3%, which were not statistically significant changes (Figure 3(a)). Additionally, hBM-MSC-CM had no effect on the cyclin-D1 expression or the apoptosis rates of MGC-803 cells (Figures 3(b) and 3(c)); however, JQ1 inhibited cyclin-D1 expression.

### 3.4. HBM-MSC-CM Promoted Migration and Glucose Uptake in MGC-803 and BGC-823 Cells

**Figure 4:**
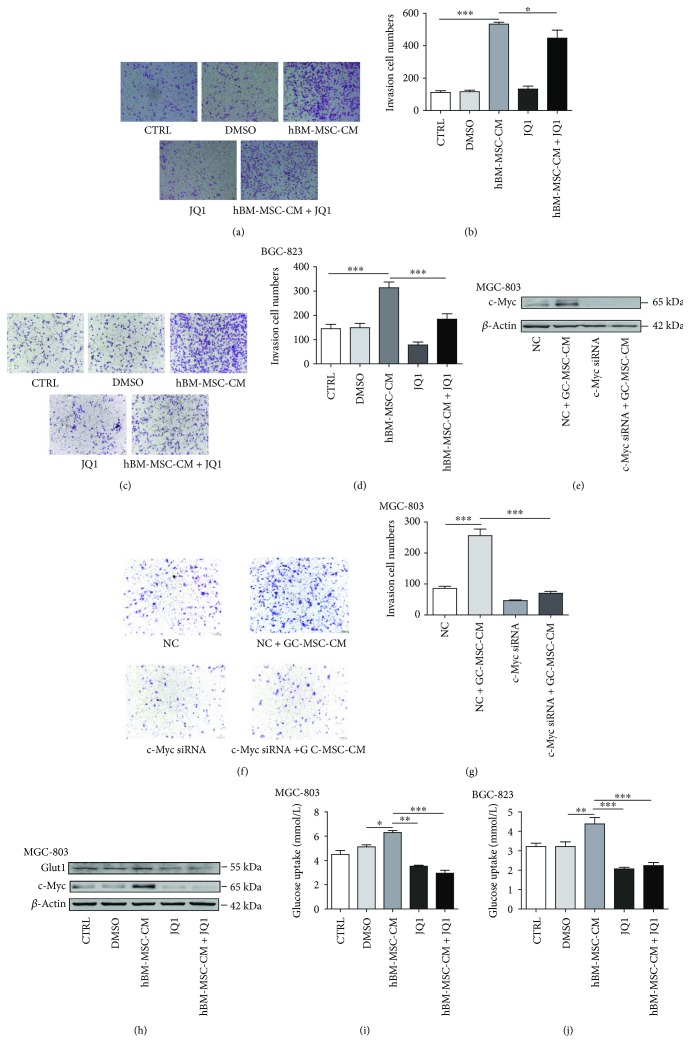
hBM-MSC-CM promoted migration and glucose uptake of MGC-803 and BGC-823 cells. (a, c) Transwell migration assays showed that hBM-MSC-CM increased the migratory ability of gastric cancer cells and that this activity was inhibited by JQ1 (scale bar: 100 *μ*m). (b, d) The numbers of migrated cells were analyzed by a one-way analysis of variance (^∗^*P* < 0.05, ^∗∗∗^*P* < 0.001). (e) c-Myc siRNA can inhibit the expression of c-Myc induced by hBM-MSC-CM in MGC-803 cells. (f, g) The effect of hBM-MSC-CM on MGC-803 cell migration can be suppressed by c-Myc siRNA (^∗∗∗^*P* < 0.001). (h) hBM-MSC-CM-induced Glut1 upregulation in MGC-803 cells was detected with Western blot. (i, j) The glucose uptake of MGC-803 and BGC-823 cells after the indicated treatments was detected using a clinical chemistry analyzer (^∗^*P* < 0.05, ^∗∗^*P* < 0.01, ^∗∗∗^*P* < 0.001).

To determine whether *in vitro* hBM-MSC-CM treatment promoted migration and glucose uptake in MGC-803 and BGC-823 cells (common features of cells with c-Myc upregulation), we performed Transwell migration, Western blot, and glucose uptake assays. Transwell migration assays showed that hBM-MSC-CM increased the migratory ability of MGC-803 and BGC-823 cells and that JQ1 significantly inhibited these effects (both *P* < 0.001; Figures 4(a)–4(d)). In order to further verify the hBM-MSC-CM promoting gastric cancer development via upregulation of c-Myc directly, we chose to use c-Myc siRNA which can inhibit c-Myc expression directly. c-Myc siRNA can inhibit the expression of c-Myc in MGC-803 cells (Figure 4(e)). Reducing the expression of c-Myc in MGC-803 cells can inhibit the effect of hBM-MSC-CM on tumor migration (Figures 4(f) and 4(g)). Western blot and glucose uptake assays also showed that JQ1 inhibited hBM-MSC-CM-induced increases in Glut1 expression in MGC-803 and glucose uptake in MGC-803 and BGC-823 cells (Figures 4(h)–4(j)).

### 3.5. HBM-MSC-CM Increased Gastric Cancer Growth *In Vivo*

**Figure 5:**
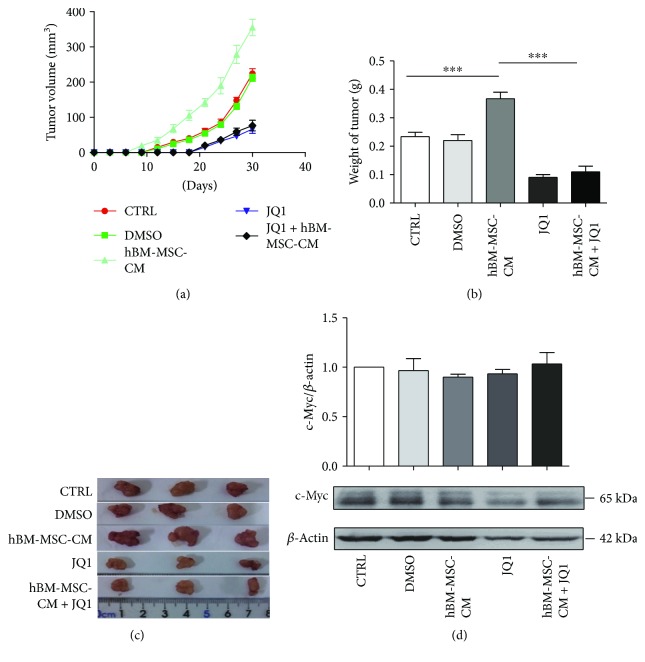
hBM-MSC-CM increased *in vivo* gastric tumor growth by upregulating c-Myc expression. (a) Tumor volumes of MGC-803 xenografts in immunocompromised mice. Tumor volumes of the JQ1 and hBM-MSC-CM + JQ1 groups were reduced compared with those of the hBM-MSC-CM and control groups; ^∗∗∗^*P* < 0.001 by Kruskal–Wallis *H* test. (b, c) Comparison of tumor weights in each group at day 30; ^∗∗∗^*P* < 0.001 by Kruskal–Wallis *H* test. (d) Expression of c-Myc in MGC-803 xenograft tissues of the different treatment groups was also detected with Western blot.

To determine whether hBM-MSC-CM promoted *in vivo* tumor growth, we injected MGC-803 cells treated with DMEM, DMSO, JQ1, hBM-MSC-CM, and hBM-MSC-CM + JQ1 for 48 h into BALB/c-nu/nu mice. Consistent with our previous studies, hBM-MSC-CM promoted tumor growth *in vivo*. Additionally, tumor volumes and weights of the JQ1 and hBM-MSC-CM + JQ1 groups were smaller than those of the hBM-MSC-CM and control groups (*P* < 0.001; Figures 5(a)–5(c)). Western blotting of xenograft tumor tissue showed that hBM-MSC-CM could not maintain the upregulation of c-Myc *in vivo* (Figure 5(d)).

## 4. Discussion

Previous studies have suggested that MSCs are closely associated with tumor progression and growth [3]; thus, there are legitimate safety concerns that limit their clinical application. Our previous study revealed that MSCs promote tumor growth *in vivo*, which suggested that these cells secrete mitogenic paracrine factors [8, 9]. Jung et al. demonstrated that the MSCs recruited by tumors facilitate metastasis [14], and Li et al. revealed that gastric cancer-derived MSCs enhanced the proliferation and migration of gastric cancer cell lines [15]. Djouad et al. reported that *in vivo* tumor growth was increased when melanoma cells were coinjected with MSCs [16]. Interestingly, Karnoub et al. found that in situ breast cancer was unchanged in the absence of MSCs; however, the migratory ability of tumor cells was significantly increased when exogenous MSCs were added [17]. In this study, we chose MGC-803 and BGC-823 cells as representative cell lines to further study the mechanism through which hBM-MSC-CM promotes tumor growth.

We explored the potential risks of using hBM-MSCs in clinical applications. Although the tumor-promoting activity of patient-derived hBM-MSCs may be more dramatic, patient-derived hBM-MSCs could not be used in clinical applications; thus, we used healthy donor-derived hBM-MSCs. Our preliminary studies showed that the mitogenic activity of hBM-MSCs on tumor cells was primarily through paracrine signaling [8]. So, we subsequently focused on the potential factors from hBM-MSCs that promoted cancer progression, as these comprised the molecular signature of potential targets that could allow their clinical application.

c-Myc is a protooncogene that is mutated in approximately 20% of human cancers and has particularly important functions in gastric carcinogenesis [18]. In the present study, we found that c-Myc expression was increased in hBM-MSC-CM-treated gastric cancer cells, suggesting that hBM-MSC-CM may promote tumor growth by upregulating c-Myc. Moreover, c-Myc has been shown to maintain normal adult stem cells and tumor stem cells [19]. It is worth noting that the effects of hBM-MSC-CM on tumor cell proliferation and cell cycle progression were not obvious *in vitro*. The experimental results showed that the upregulation of c-Myc expression in tumor cells could maintain 12 hours after withdrawal of hBM-MSC-CM. However, the inhibitory effect of JQ1 on tumor cells was clear in this setting. Our previous studies have shown that hBM-MSCs promote tumor angiogenesis through paracrine signaling, and Rahl et al. found that upregulating c-Myc also promotes tumor angiogenesis [20]. After pretreatment of tumor cells, the upregulation of c-Myc induced by hBM-MSC-CM cannot be maintained for a long time. Though the upregulation of c-Myc in tumor tissue was not maintained, the upregulation of c-Myc in tumor cells has the effect of initiating tumor development in mice. HBM-MSC-CM upregulated the expression of c-Myc in tumor cells, which may lead to reprogramming of tumor cells and affect tumor growth by promoting angiogenesis or the increase of glycolysis *in vivo*. This effect does not depend on the long-term expression of c-Myc. We now hypothesize that hBM-MSC-CM promotes tumor angiogenesis via upregulation of c-Myc, and thus, there are both cell-intrinsic and cell-extrinsic *in vivo* effects of hBM-MSC-CM that increase tumor growth. We will continue to explore these processes in future studies. In conclusion, our data show that hBM-MSC-CM in the tumor microenvironment increases c-Myc expression in tumor cells, which directly promotes tumor cell proliferation and increases vascularization.

JQ1 is a small selective bromodomain and extraterminal (BET) motif inhibitor that can inhibit c-Myc expression and tumor growth [21, 22]. Other studies have reported that a critical mechanism of JQ1 in suppressing tumor growth is its ability to block c-Myc expression [23–26]. We found that JQ1 inhibits the expression of c-Myc. Moreover, hBM-MSC-CM promoted glucose uptake and cell migration via upregulating c-Myc in gastric cancer, both of which were suppressed by JQ1. Finally, *in vivo* studies showed that JQ1 inhibited the increased growth of gastric cancer xenografts that was induced by hBM-MSC-CM. Clinical trials have proven that hBM-MSCs do not undergo tumorigenesis [4, 6]. But tumor occurrence is the result of variety of tumor-promoting factors. However, BM-MSCs are immunosuppressive, and factors that affect the immune system should be a concern with regard to tumorigenesis [27, 28]. In the present study, we found that hBM-MSC-CM can promote tumor cell proliferation, migration, and glucose uptake via upregulating c-Myc. Although there are no reports of hBM-MSCs causing tumor formation, their potential to promote tumorigenesis is worthy of concern for potential applications in tissue engineering.

## 5. Conclusions

hBM-MSC-CM promotes gastric cancer cell proliferation by upregulating c-Myc. c-Myc inhibitors may be effective at preventing the protumor effects of hBM-MSC-CM and, therefore, could solve the clinical safety issues of hBM-MSCs.

## Data Availability

The data used to support the findings of this study are included within the article.

## Abbreviations

MSCs: Mesenchymal stem cells
hBM-MSCs: Human bone marrow MSCs
hBM-MSC-CM: hBM-MSC-conditioned medium
BRDs: Bromodomain-containing proteins
PBS: Phosphate-buffered saline
FBS: Fetal bovine serum
DMEM: Dulbecco's modified Eagle's medium
BET: Bromodomain and extraterminal motif.
